# The effectiveness of high-intensity CBT and counselling alone and following low-intensity CBT: a reanalysis of the 2nd UK National Audit of Psychological Therapies data

**DOI:** 10.1186/s12888-018-1899-0

**Published:** 2018-10-03

**Authors:** Michael Barkham, David Saxon

**Affiliations:** Department of Psychology, University of Sheffield, Sheffield, UK

## Abstract

**Background:**

A previously published article in this journal reported the service effects from 103 services within the UK Improving Access to Psychological Therapies (IAPT) initiative and the comparative effectiveness of CBT and Counselling provision. All patients received High-intensity CBT or High-intensity Counselling, but some also received Low-intensity CBT before being stepped-up to High intensity treatments. The report did not distinguish between patients who received low-intensity CBT before being stepped-up. This article clarifies the basis for collapsing low- and high-intensity interventions by analysing the four treatment conditions separately.

**Method:**

Data from 33,243 patients included in the second round of the National Audit of Psychological Therapies (NAPT) were re-analysed as four separate conditions: High-intensity CBT only (*n* = 5975); High-intensity Counselling only (*n* = 3003); Low-intensity CBT plus High-intensity CBT (*n* = 17,620); and Low-intensity CBT plus High-intensity Counselling (*n* = 6645). Analyses considered levels of pre-post therapy effect sizes (ESs), reliable improvement (RI) and reliable and clinically significant improvement (RCSI). Multilevel modelling was used to model predictors of outcome, namely patient pre-post change on PHQ-9 scores at last therapy session.

**Results:**

Significant differences obtained on various outcome indices but were so small they carried no clinical significance. Including the four treatment groups in a multilevel model comprising patient intake severity, patient ethnicity and number of sessions attended showed no significant differences between the four treatment groups. Comparisons between the two high-intensity interventions only (*N* = 8978) indicated Counselling showed more improvement than CBT by 0.3 of a point on PHQ-9 for the mean number of sessions attended. However, this result was moderated by the number of sessions and for 12 or more sessions, the advantage went to CBT.

**Conclusions:**

This re-analysis showed no evidence of clinically meaningful differences between the four treatment conditions using standard indices of patient outcomes. However, a differential advantage to high-intensity Counselling for fewer than average sessions attended and high-intensity CBT for more than average sessions attended has important service implications. The finding of equivalent outcomes between high-intensity CBT and Counselling for more severe patients also has important policy implications. Empirically-informed procedures (e.g., predictive modelling) for assigning patients to interventions need to be considered to improve patient outcomes.

**Electronic supplementary material:**

The online version of this article (10.1186/s12888-018-1899-0) contains supplementary material, which is available to authorized users.

Recently, this journal reported the outcomes of the 2nd National Audit of Psychological Therapies [1]. The report focused on the data drawn from services within the UK government's Improving Access to Psychological Therapies (IAPT) initiative. IAPT services use a stepped care model where a majority of patients are initially treated at step 2 with low-intensity (Li) CBT-based interventions. Patients with depression who do not respond are stepped-up to a high-intensity (Hi) step 3 therapy, predominantly CBT or Counselling. Patients deemed to be more severe may be stepped-up directly to a step 3 therapy. Hence, for patients who receive a high-intensity intervention, there are 4 possible options: Low-intensity CBT + High-intensity CBT, (Li-CBT/Hi-CBT), Low-intensity CBT + High-intensity Counselling (Li-CBT/Hi-Counselling), High-intensity CBT only (Hi-CBT), and High-intensity Counselling only (Hi-Counselling).

The previous report [1] focused on service-level effects from 103 services and, in order to maximise the power of treatment comparisons, defined the two therapies according to the high-intensity format as all patients received this form either immediately or following a low-intensity CBT intervention. Not reported in the original publication were the percentages of patients receiving step 2 followed by step 3 therapy, and those receiving only step 3 (i.e., high-intensity) therapy. For CBT the percentages were 74.7% for Li-CBT/Hi-CBT (step 2 and 3) and 25.3% for Hi-CBT (step 3 only); and for Counselling the percentages were 68.9% for Li-CBT/Hi-Counselling (step 2 and 3) and 31.1% for Hi-Counselling (step 3 only). We found no meaningful differences between the four interventions. However, that analysis and explication was not included in the original report.

The primary aim of this report was to present a more refined analyses comparing CBT and Counselling outcomes in terms of the four types of treatment episodes as opposed to collapsing low and high-intensity deliveries of each modality of therapy as in the previous report.

## Method

The study sample, 33,243 patients treated at 103 IAPT sites, was the same as that used in the original article. It comprised a subsample of the data collected from 220 services as part of the second audit of all NHS-funded psychological therapy services for adults in primary and secondary care in England and Wales [2].

As reported above, the majory of patients were initially allocated to low-intensity CBT. Patients were allocated to high-intensity therapy through standard routine practice procedures either directly, based on need, or via stepping up from low-intensity CBT. Such decision rules vary across services but will include availability of a practitioner regardless of their theoretical orientation, assignment by a step 2 practitioner in terms of the issues identified by the patient (e.g., relationship issues being assigned to counselling and specific problems being assigned to CBT), or patient stated preferences.

Outcome was change in PHQ-9 [3] scores from the start of treatment episode to the last treatment session of the high-intensity therapy. As in the original analysis, multilevel modelling (MLM) and Markov chain Monte Carlo (MCMC) procedures were used to model the nested structure of patients within services and to control other variables. Variable coefficients were considered significant if they were more than 1.96 times their standard errors [4, 5]. Further analysis considered levels of reliable and clinically significant improvement (RCSI) for the four treatments conditions [6, 7].

## Results

Table 1 presents the four treatment conditions in terms of the severity of patients at intake and their outcomes, the number of sessions attended, and effect sizes. There were small but statistically significant differences between the four conditions for intake severity in terms of pre-therapy PHQ-9 score (ANOVA: *F* (3, 33,239) = 14.38. *p* < 0.001) and the proportion of clinical patients at intake assessment (χ^2^ = 53.31, *p* < 0.001). Pairwise comparisons in ANOVA showed patients receiving Hi-Counselling to be less severe than the three other conditions (all *p*-values < 0.001), but also that patients were more severe at the start of Li-CBT/Hi-Counselling than at the start of Li-CBT/Hi-CBT (*p* = 0.020).

**Table 1:** The four patient groups described according to total numbers and numbers at a clinical level pre-treatment, pre-treatment PHQ-9, pre-post PHQ-9 change and effect size, sessions attended, patient change per session, and percentage meeting criteria for reliable and clinically significant improvement

Patient group	N (%)	Clinical at pre- treatment N (%)	Pre–PHQ-9 Score Mean (SD)	Pre-Post change Mean (SD)	Pre-post effect size (95% CI)	Sessions attended Mean (SD)	Patient change on PHQ-9 per session Mean (SD)	RCSI rate % (95% CI)
Li-CBT/Hi-CBT	17,620 (53.0)	14,069 (80.0)	15.4 (6.49)	6.1 (6.95)	0.94 (0.92, 0.96)	9.0 (6.40)	0.91 (1.48)	52.1 (51.3, 52.9)
Li-CBT/Hi-Counselling	6645 (20.0)	5459 (82.2)	15.6 (6.20)	6.1 (6.83)	0.98 (0.95, 1.02)	8.0 (5.94)	1.03 (1.56)	50.2 (48.9, 51.6)
Hi-CBT	5975 (18.0)	4719 (79.0)	15.4 (6.58)	5.9 (6.98)	0.90 (0.86, 0.93)	8.8 (6.14)	0.85 (1.43)	49.4 (47.9, 50.8)
Hi-Counselling	3003 (9.0)	2280 (75.9)	14.7 (6.60)	5.5 (6.63)	0.83 (0.78, 0.89)	6.4 (4.74)	1.08 (1.74)	49.2 (47.1, 51.2)
Overall	33,243	26,527 (79.8)	15.4 (6.47)	6.0 (6.91)	0.93 (0.91, 0.94)	8.5 (6.18)	0.94 (1.52)	51.0 (50.4, 51.6)

Note: *Li-CBT/Hi-CBT* Low-intensity CBT stepped up to high-intensity CBT, *Li-CBT/Hi-Counselling* Low-intensity CBT stepped up to high-intensity Counselling, *Hi-CBT* High-intensity CBT only, *Hi-Counselling* High-intensity Counselling only

### Pre-post change

Comparing pre-post change on the PHQ-9, controlling for intake PHQ-9 scores, an ANCOVA indicated a significant difference between treatment groups overall (*F* (3, 33,238) = 3.43, *p* = 0.016). In comparing the four groups, the only significant differences were between Li-CBT/Hi-CBT and both Hi-CBT (*p* = 0.032) and Hi-Counselling (*p* = 0.006). However, the differences in pre-post change in all comparisons were small; 0.20 and 0.34 of a PHQ-9 point respectively. There were no significant differences in other comparisons (all *p*-values between 0.084 and 0.355). Comparing effect sizes with 95% CIs showed no significant differences between CBT and Counselling when preceded by Li-CBT, and both groups had a larger effect than Hi-Counselling, while Li-CBT/Hi-Counselling also had a larger effect than Hi-CBT. There was no significant difference between Hi-CBT and Hi-Counselling.

The RCSI rates also showed significant differences (χ^2^ = 16.06, *p* = 0.001). However, the 95% CIs of the rates overlapped apart from the comparison between Li-CBT/Hi-CBT and both Hi-Counselling and Hi-CBT, with Li-CBT/Hi-CBT having a significantly better RCSI rate.

### Change per session

Hi-Counselling had significantly fewer treatment sessions than the other three treatment groups (M-W U: *p* < 0.001 in each comparison). Li-CBT/Hi-Counselling also had fewer sessions than Li-CBT/Hi-CBT (M-W U: *p* < 0.001). As a result, the mean patient change per session was greater for the two groups with a Hi-Counselling component. The difference in change per session between the four groups was significant (K-W: *p* < 0.001), with pairwise comparisons indicating significant differences between each Counselling group and both the Li-CBT/Hi-CBT and the Hi-CBT groups (M-W U: *p* < 0.001 in all four comparisons) and a significant difference between the two CBT groups (M-W U: *p* = 0.002). However, there was no significant difference in change per session between the Li-CBT/Hi-Counselling and the Hi-Counselling groups (M-W U: *p* = 0.203).

### Comparisons between the four groups

Including the four treatment groups in a multilevel model (see Additional file 1) that comprised patient intake severity, in terms of PHQ-9 and GAD-7 scores, patient ethnicity and number of sessions attended indicated no significant differences between the outcomes for Hi-Counselling (the reference group in the model) compared to the other three treatment groups. However, the interaction between treatment group and sessions indicated a significant difference between Hi-Counselling and Hi-CBT in how the number of sessions attended moderated the treatment effect. More sessions generally improved outcomes, but for each session above the average number (i.e., > 9 sessions), Hi-CBT improved outcomes by 0.106 of a point on PHQ-9 more than Hi-Counselling. For each session less than average (i.e., < 9 sessions), Hi-Counselling was more effective by the same amount. A similar result obtained between Hi-Counselling and Li-CBT/Hi-CBT, although the difference was less (0.075 of a point on PHQ-9 for each session).

### High-intensity comparisons

Replicating the multilevel model with only those patients receiving a high-intensity intervention (*N* = 8978) indicated that Hi-Counselling was more effective than Hi-CBT when controlling for intake severity on PHQ-9 and GAD-7, ethnicity, and number of sessions attended. Overall, Hi-Counselling showed more improvement than Hi-CBT by 0.3 of a point on PHQ-9 for the average number of sessions attended (8 sessions in this sample). However, this was moderated by the number of sessions attended with each session below average increasing this difference by 0.1 of a point and each session above average reducing the difference by the same amount such that at 12 or more sessions, CBT was more effective.

### Comparisons for moderate-severe and severe patients

A greater proportion of Hi-CBT patients were severe at intake (PHQ-9 > 20), 31.7% compared with 26.7% (χ^2^ = 28.95, *p* < 0.001) but the rates were similar for moderate-severe patients (PHQ-9: 15–19): 26.9% compared with 26.8% respectively. In terms of outcomes, there were no significant differences between Hi-CBT and Hi-Counselling in pre-post change for severe (ANCOVA: F (1, 2693) = 0.33, *p* = 0.566) or moderate-severe (ANCOVA: F (1, 2409) = 0.103, *p* = 0.749) patients. Similarly, there were no significant differences between the treatments in terms of the percentage of severe or moderate-severe patients obtaining threshold for reliable improvement and the more stringent RCSI index. For example, for severe patients, reliable improvement rates (with 95% CIs) were: Hi-CBT, 61.4% (59.2, 63.6); Hi-Counselling, 61.5% (58.0, 64.9).

## Discussion

The findings from this 4-way analysis are consistent with the earlier reported results in showing broad equivalence in outcomes between patients who received CBT-based and Counselling-based interventions. However, Hi-Counselling was slightly more effective with shorter term treatment while Hi-CBT was slightly more effective with longer term treatment. The current results showed that this was the case whether or not patients had low-intensity CBT prior to either Hi-CBT or Hi-Counselling. This finding raises questions about why and how patients are stepped up at different services and how this stepping up procedure could be more ‘evidence-based’ and consistent in order to improve outcomes for both step 3 therapies. In this respect, results from studies applying predictive modelling to outcomes from comparative trials [8] and IAPT services [9] appear to be a promising way forward in terms of moving towards personalised treatments and the possibility of raising the improvement rates for patients rather than privileging one therapy model over another.

A Li-CBT intervention prior to Hi-Counselling appeared to add little to the outcomes of Counselling whereas when followed by Hi-CBT, outcomes were improved. However, clinical differences were small between treatments, amounting to fractions of a single point on the PHQ-9. Indeed, this was an overall observation from this 4-way reanalysis, namely that such differences that did occur were of doubtful clinical significance.

It was a limitation in the dataset that the number of sessions for step 2 and step 3 phases separately were not known. However, our analysis on a restricted sample of patients who only received a step 3 high-intensity treatment indicated that CBT and counselling outcomes did not differ, a finding consistently reported in the literature [10].

Overall, the findings reported in this article extend findings from the previous report [1] in showing that differences between the four treatments were small and varied as a function of which index was used. However, for high-intensity treatments only, Counselling showed a small advantage over CBT but only for treatment durations up to 11 sessions. Where treatment duration was 12 sessions or more, CBT showed a small advantage. Such a result should be of interest to service providers and commissioners where cost implications are a factor. It also adds to the previous report in showing that high-intensity Counselling is equally as effective as CBT in treating more severe depression. Such a finding challenges the current NICE guideline for the management of severe depression in which Hi-Counselling is not recommended for patients presenting with severe depression [11].

## Additional file

Additional file 1: Multilevel model of PHQ-9 change using Markov chain Monte Carlo (MCMC). (DOCX 44 kb)

## References

[1] Pybis J, Saxon D, Hill A, Barkham M (2017). The comparative effectiveness and efficiency of cognitive behaviour therapy and counselling in the treatment of depression: evidence from the 2^nd^ UK National Audit of Psychological Therapies. BMC Psychiatry.

[2] Report of the second round of the National Audit of Psychological Therapies. Royal College of Psychiatrists. 2013. https://www.rcpsych.ac.uk/pdf/NAPT%20second%20round%20National%20report%20%20website%2028-11-13v2.pdf. Accessed 31 May 2018.

[3] Kroenke K, Spitzer RL, Williams JB (2001). The PHQ-9: validity of a brief depression severity measure. J Gen Intern Med.

[4] Snijders TAB, Bosker RJ (2012). Multilevel analysis: an introduction to basic and advanced multilevel modelling.

[5] Rasbash J, Charlton C, Browne WJ, Healy M, Cameron B, Version MLN. 2.1. Centre for Multilevel Modelling. University of Bristol; 2009.

[6] Evans C, Margison F, Barkham M (1998). The contribution of reliable and clinically significant change methods to evidence-based mental health. Evid Based Ment Health.

[7] Jacobson NS, Truax P (1991). Clinical significance: a statistical approach to defining meaningful change in psychotherapy research. J Consult Clin Psychol.

[8] Huibers MJH, Cohen ZD, Lemmens LHJM, Arntz A, Peeters FPML, Cuijpers P, DeRubeis RJ (2015). Predicting optimal outcomes in cognitive therapy or interpersonal psychotherapy for depressed individuals using the personalized advantage index approach. PLoS One.

[9] Delgadillo J, Huey D, Bennett H, McMillan D (2017). Case complexity as a guide for psychological treatment selection. J Consult Clin Psychol..

[10] Stiles WB, Barkham M, Mellor-Clark J, Connell J (2008). Effectiveness of cognitive-behavioural, person-centred, and psychodynamic therapies in UK primary care routine practice: replication in a larger sample. Psychol Med..

[11] NICE. Depression in adults: The treatment and management of depression in adults. NICE clinical guideline 90; 2009. https://www.nice.org.uk/guidance/CG90. Accessed 31 May 2018.

