# Supplementary material for: The effectiveness of high-intensity CBT and counselling alone and following low-intensity CBT: a reanalysis of the 2nd UK National Audit of Psychological Therapies data
Source: BMC Psychiatry. 2018 Oct 3;18:321. doi: 10.1186/s12888-018-1899-0 (PMC6171289; doi:10.1186/s12888-018-1899-0)
Supplement: Supplementary file 1 — Multilevel model of PHQ-9 change using Markov chain Monte Carlo (MCMC). (DOCX 44 kb) [file 12888_2018_1899_MOESM1_ESM.docx]

Final MCMC model

Appendix


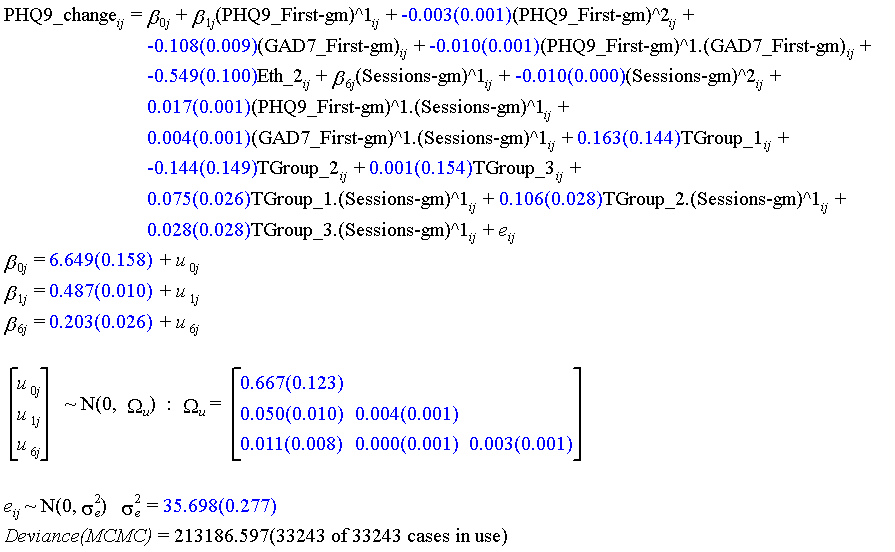


TGroup_1: Li- CBT/Hi-CBT; TGroup_2: Hi-CBT only; TGroup_3: Li-CBT/Hi-Counselling. Reference category is Hi-Counselling only. Eth_2: Ethnic minority patients. Continuous variables are grand mean (gm) centred.
